# Mathematical Methods and Algorithms for Improving Near-Infrared Tunable Diode-Laser Absorption Spectroscopy

**DOI:** 10.3390/s18124295

**Published:** 2018-12-06

**Authors:** Tianyu Zhang, Jiawen Kang, Dezhuang Meng, Hongwei Wang, Zhengming Mu, Meng Zhou, Xiaotong Zhang, Chen Chen

**Affiliations:** Key Laboratory of Geophysical Exploration Equipment, Ministry of Education, College of Instrumentation & Electrical Engineering, Jilin University, Changchun 130026, China; zty@jlu.edu.cn (T.Z.); kangjw6515@mails.jlu.edu.cn (J.K.); mengdz6515@mails.jlu.edu.cn (D.M.); wanghw6515@mails.jlu.edu.cn (H.W.); muzm17@mails.jlu.edu.cn (Z.M.); zhoumeng17@mails.jlu.edu.cn (M.Z.); zxt18@mails.jlu.edu.cn (X.Z.)

**Keywords:** TDLAS, signal processing, gas sensor, denoise, interference fringe, background correction

## Abstract

Tunable diode laser absorption spectroscopy technology (TDLAS) has been widely applied in gaseous component analysis based on gas molecular absorption spectroscopy. When dealing with molecular absorption signals, the desired signal is usually interfered by various noises from electronic components and optical paths. This paper introduces TDLAS-specific signal processing issues and summarizes effective algorithms so solve these.

## 1. Introduction

Sensors based on Tunable Diode Laser Absorption Spectroscopy (TDLAS) have the advantages of high sensitivity, high stability, high selectivity and fast response, and have been widely applied in atmospheric environmental monitoring [1,2,3], medical health [4], industrial production [5,6], military surveying [7,8] and other fields. Given that the absorption spectrum is primarily determined by the atomic and molecular composition of the measured sample, it is a useful tool to determine the presence of a particular substance in a sample [9,10,11,12]. Nevertheless, the performance of TDLAS systems can be limited by many factors [13,14], especially, the measurement signal incorporates numerous contributions from optical components (interference fringe) and electronic components [15,16]. Background errors, which are caused by background extraction, also limit the detection precision of the system. Therefore, signal preprocessing is necessary to improve the accuracy of TDLAS-based analytical instruments.

In the development of TDLAS, many methods have been proposed to improve system accuracy and to measure resolution [17,18,19,20,21,22], which can be divided into two classes: software processing and hardware-based processing. Some hardware-based approaches such as multi-pass cells, differential schemes, and wavelength modulation techniques have been proved to ameliorate signal quality effectively [23,24,25,26]. Besides, with the continuous evolution of data processing technology, many data analysis algorithms have emerged in some fields [27,28]. Some of these methods have been introduced and applied in TDLAS systems to enhance the accuracy and resolution performance. Thereby the current review focuses more on software-based methods. Some algorithms have also been proposed to address pertinent issues in TDLAS signals, and the applicability of these algorithms have been experimentally demonstrated.

## 2. Factors Affecting the Accuracy of TDLAS

**Figure 1:**
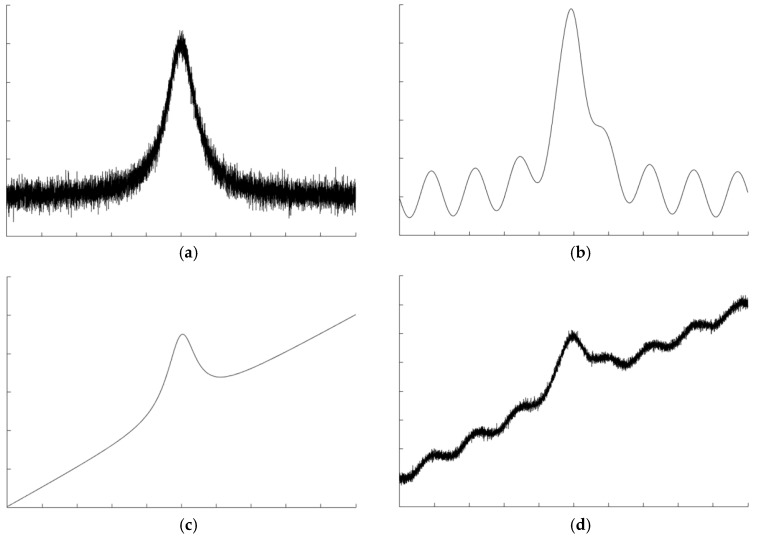
Signal simulation. (**a**) White noise; (**b**) interference fringe; (**c**) background; (**d**) multiple interference signal.

Many factors can affect the accuracy of a TDLAS system [29], such as white noise in electronic units, interference fringes in optical components [30,31], errors in background extraction [32,33], signal drift, and atmospheric pressure changes [34,35]. Some errors even arise from dust in light paths [36,37] and mechanical jamming when the device is used. Among these errors, the first three interferences are universal, and this article mainly introduces research works on these three issues. Examples of these polluted waveforms are shown in Figure 1.

Among these problems, the most common issue is background error, which is easier to work out but should be given priority to deal with. Interference fringes are relatively difficult to remove, and are usually the main constraint on the accuracy of TDLAS. The noise, especially high-frequency white noise, should be handled after the first two problems are resolved.

### 2.1. Denoise

The signal obtained from the TDLAS system usually tends to exhibit a limited signal-to-noise ratio (SNR). The amplitude of absorption peak is smaller than that in other detection methods because the gas absorption is weaker in the near-infrared than in the mid-infrared region [38]. Especially in trace gas detection, the gas absorption signal is so weak that it can be easily submerged in various noise and spikes. These noises may originate from TEC or lock-in amplifier, the 1/f noise or power supply voltage jitter [39]. In this case, those algorithms that are most commonly used in general signal processing, like the least-squares method, cannot efficiently extract the precise desired signal. In recent years, some advanced algorithms have been proposed or introduced into TDLAS systems to process signals with low SNR, including wavelet transform (WT) [39,40], adaptive Savitzky–Golay algorithm [41], and empirical mode decomposition (EMD)-FCR algorithm [42]. These algorithms have been verified to be significant for practical issues.

### 2.2. Interference Fringe

The detection sensitivity of TDLAS technology is severely restricted by optical interference due to the strong coherence of the laser [43,44]. Especially at low concentration, this disturbance leads to baseline fluctuation and causes error in waveform extraction [45]. These optical interferences may arise from the multiple reflections on the reflecting or scattering surfaces in the light path [43,46], which then periodically fluctuates like a sine function in the measurement signal. Unlike the electrical noise, optical interference like the effect of interference fringes is in the low frequency segment and exhibits large amplitude. Distinguishing the location of signal peaks under some extreme cases may be difficult. Even if a comprehensive algorithm, such as the WT, can perform interference fringe removal, it cannot achieve high-precision extraction in many special situations. Some strategies for removing optical interference have been proposed [47,48], and these targeted algorithms give better results compared with common methods.

### 2.3. Background Correction

The carrier density inside the diode laser controls the light emission frequency and light emission intensity equally due to the nature of the semiconductor laser. Hence, when the modulation current drives the laser to sweep the absorption peak of the gas, the incident laser intensity will also be similarly modulated. As a result, the measuring signal peak will contain a ramp known as “background”. Before extracting useful information, the raw spectral signal must be intensity normalized, that is, the raw spectrum is divided by the background line. For the information contained in the measurement signal, the error caused by the background removal also causes an error in the peak and outline area of the signal waveform, especially for the method of calculating the concentration information by using the signal area. The error is relatively large. Therefore, the background correction pretreatment is quite necessary. Like the removal of optical interference, background correction can be performed by some comprehensive algorithms, such as the EMD algorithm, which can obtain some background information by decomposing a final Intrinsic Mode Function (IMF) [49,50]. Some improvements that add an iteration process have been proposed to achieve an accurate background correction [51,52], and the ideas and strategies in these algorithms can be referenced to address the background problem before other signal processes.

## 3. Algorithms for TDLAS Signal Processing

Algorithms applied to denoising and signal processing have been further developed, and signal averaging is an early and simple method for signal processing [53]. Many kinds of algorithms have been developed to denoise from different perspectives, examples of which are the linear filters based on early filtering theory, such as the Wiener filter and Kalman filter [54,55]; the fitting algorithms based on nonlinear regression, such as least-squares method; and some other decomposition algorithms based on signal decomposition and reconstruction, such as empirical mode decomposition [49]. In recent years, some novel strategies have been developed to deal with the usual signal disturbances in TDLAS. For any denoising process, the ideal situation is to obtain a priori knowledge of a noise model before selecting an algorithm. Therefore, algorithm classification based on noise models has practical significance. This article will introduce some solutions addressing the three issues mentioned above. To compare the different methods, we present the experimental performance of each algorithm in each section.

### 3.1. Denoising

Noise reduction is required in various signal preprocessing techniques. In this section the particular strengths of the WT, adaptive Savitzky–Golay algorithm, and EMD-FCR algorithm will be introduced for their applications in TDLAS noise deduction. In addition to white noise removing, these methods can theoretically solve other interferences to some extent. In some studies, WT [56] is introduced into TDLAS sign analytical process, which has been widely used in other signal processing fields [16]. Wavelet analysis is a signal time-frequency analysis method for processing local or transient signals. It originates from Fourier algorithm transformation, combines the concepts of signal stretching and translation, and involves dual locality in the time and frequency domains. This variable resolution analysis method focuses on both the low-frequency trends and high-frequency details of signals.

#### 3.1.1. Wavelet Transform (W-T)

**Figure 2:**
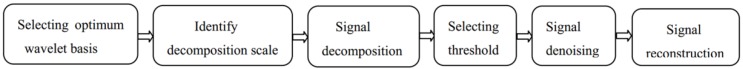
Flow chart of WT. This figure was obtained from reference [39].

The wavelet-based scenarios can be an effective approach to modeling the absorption and work out complicated signal situations because of the special characteristics of the time-frequency relationship. The amplitude of the absorption signal peak is small and contains both high-frequency white noise and low-frequency fluctuations which are usually caused by temperature drift and interference fringes. WT offers a window that varies with signal frequency band, allowing different scales of noise to be resolved into different sub-bands. As a result, time resolution improves at high frequency and frequency resolution improves at low frequency [57,58,59]. Thus, wavelet denoising is a powerful tool to extract desired signals from multiple noise pollution. However, one of the drawbacks of WT is it’s the strong subjectivity of the choice of parameters. Human errors will greatly affect the decomposition performance of the algorithm. In this part, we mainly introduce the studies of WT to deal with the noise of low-SNR situation, in which the experimental process and data show significant reference for further research, and the background removal application will be introduced later in this article. In this paper, only the key concepts of WT are presented. Detailed mathematical treatment can be found in the cited references. Compared with e Fourier transform (FT), WT uses a finite-length, attenuating wavelet basis as a decomposition basis function, instead of an infinite-length trigonometric function. By selecting different scale functions and wavelet basis, it is possible to synthesize signals with time-domain scale discrepancy. Similarly, when decomposing a signal by wavelet basis and decomposition scale, its localization characteristics can be mapped into different frequencies. Thus, this method can be used to analyze non-stationary signals. Furthermore, by choosing an appropriate threshold to filter the decomposition result and reconstruct this signal, undesired noise can be removed, which is referred to as wavelet denoising (WD). The flow chart of the WT is shown in Figure 2.

**Figure 3:**
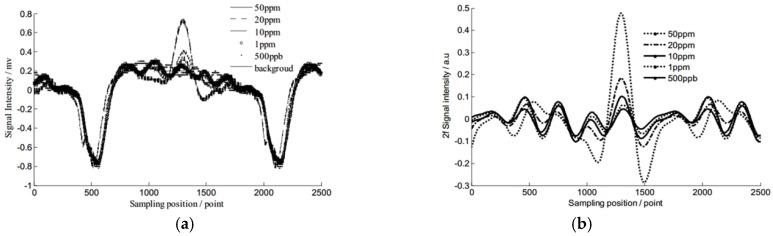
(**a**) signal without WD (**b**) signal with WD. This figure was obtained from reference [39].

Xia et al. [39] introduced WT to deal with low-SNR signal in TDLAS systems. In their experiments, wavelet basis symlet 6 (a kind of symmetric basis function) and decomposition scale six was used, and an approximation coefficient of a term of absorption signal was reserved and reconstructed only at a certain frequency. After signal reconstruction, the signal without and with WD was compared. Figure 3 shows that wavelet denoising can strikingly optimize the signal.

Zheng et al. [40] also studied the application of wavelet-denoising-assisted wavelength modulation technique in a TDLAS-based near-infrared CH_4_ detection device. Furthermore, detailed experimental data are provided to confirm the improvement of WD for polluted signals. A comparison between the sensing performances under the cases with and without WD use is shown in Table 1. Moreover, the sample gas was set up in two groups, a low-concentration group (scale of 0–1 kppm) and a high-concentration group (scale of 0–50 kppm). Experimental results demonstrated that the wavelet denoising method has great practical significance, and especially in low-concentration gas detection, the quality of the signal is enhanced significantly.

#### 3.1.2. Adaptive Savitzky–Golay (S–G) Algorithm

S–G filter is a classic smoothing denoising method [60,61] and is one of the most common pretreatment methods in spectrum analysis [62]. Li et al. proposed a simple but robust modified adaptive S–G algorithm for TDLAS signal processing [41], which shows unique superiority when temporal resolution and low system cost are priorities. This approach is developed from the S–G smoothing filter. The S–G filter using the least squares fitting coefficient as a filter response function is a smoothing filtering method for high frequency noises. As for the basic S–G filter, its effectiveness is strongly dependent on window size. As explained above, one of the difficulties in TDLAS signal processing is that the noise can originate from multi-frequency components. With a fixed window it is hard to match each of these signal segments. This new method presents a variable window and provides two additional criteria for TDLAS signal processing to determine the optimal window size. Compared with many preset parameters of WT, this adaptive algorithm reduces the subjective error.

**Figure 4:**
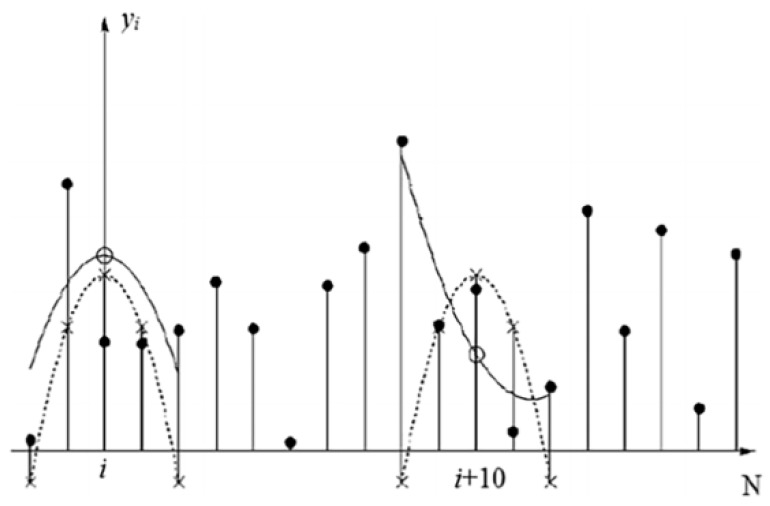
Illustration of least-squares smoothing by locally fitting a low-order polynomial (solid line) to five input samples: dot denotes the raw input samples, circle denotes the least-squares smoothed samples, and x denotes the effective impulse response samples. The dotted line denotes the polynomial approximation to centered unit impulse. This figure was taken from [41].

The basic method of the S–G algorithm involves the following steps: (i) selecting window size (ii) selecting a polynomial function for the data point in window (iii) correcting the data point at the center of the selected interval by the polynomial coefficients as shown in Figure 4 and shifting the analysis window to the right by one data point. 

**Figure 5:**
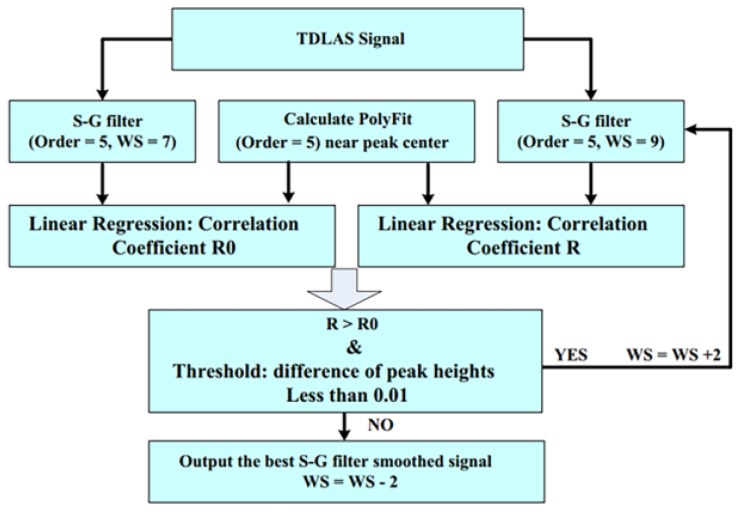
Flow chart of adaptive Savitzky–Golay algorithm. This figure was adopted from [41].

The above process is repeated. In this modified approach, two criteria are introduced to work out the optimal window size, namely, “PolyFit” and a threshold “Th”. “PolyFit” is a signal segment in a polynomial function, which we regarded as noiseless. In a process of a segment of data, correlation coefficient R between the “PolyFit” and the same segment in the S–G-filter-smoothed data is utilized to assess the optimal filtering parameters instead of SNR. This condition is valid for noise reduction but is not credible for signal preservation. The threshold “Th”, which is defined as the difference of peak heights between “PolyFit” and the S–G filtering smoothed data, is used to ensure filtering parameters without excessive signal distortion. Thus, each data interval can be modified under an optimal window size, and potential signal distortion can be alleviated in signal processing. The flow chart of the modified algorithm is shown in Figure 5.

A series of experiments was performed to investigate the effectiveness of the algorithm and its applicability in various situations, for example, suitability evaluation for absorption spectra with different line shapes under the different pressures (between a few mbar and 1 bar). These experimental results indicated that the developed algorithm is reliable for practical application, and this method could also be used to construct an optimal calibration model for TDLAS spectra with different background structural characteristics (linear or nonlinear baseline drift). However, when applying the method to the simulated signals with different sampling points, one has to compromise between noise reduction and temporal resolution.

**Figure 6:**
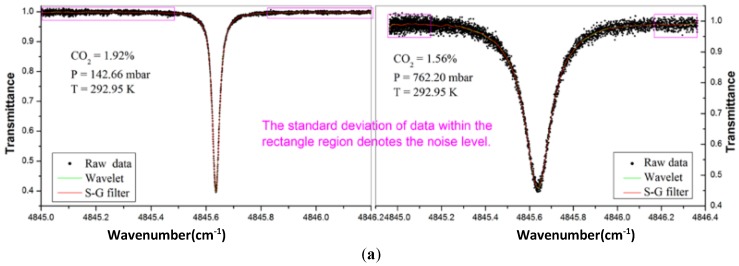

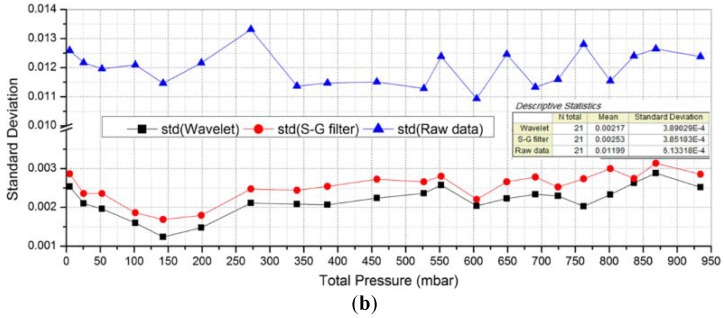
(**a**) Raw signal and processed signal by wavelet and S–G filter under different noise level. (**b**) Standard deviations of three sets of data under different pressure. This figure was obtained from [41].

At a concentration of 1.5% of CO_2_, the filter results of S–G algorithm compared with WT-based filter are shown in Figure 6a,b. The WT-based filter shows a strong noise reduction ability, the SNR enhancement factor is 5.5, and the S–G filter is 4.7. However, the WT-based filter requires more parameters and costs more time.

#### 3.1.3. EMD-FCR Algorithm

EMD algorithm is a time domain decomposition method based on the time scale features of the processed data [63]. EMD has been widely applied in many fields due to its excellent performance in processing non-stationary and non-linear signals [64,65,66,67]. In theory, the EMD algorithm can decompose any complicated signal into finite IMFs, and preset basis functions are not required. The signal decomposition depends only on the characteristics of signal itself, which is the essential difference from WT. Meng et al. [42] introduced the EMD algorithm into TDLAS signal processing, and proposed an improved algorithm that combines EMD, S–G filter, cross-correlation, and signal reconstruction (FCR), which is referred to as the EMD-FCR algorithm. This new method shows better applicability for second harmonic signal processing.

**Figure 7:**
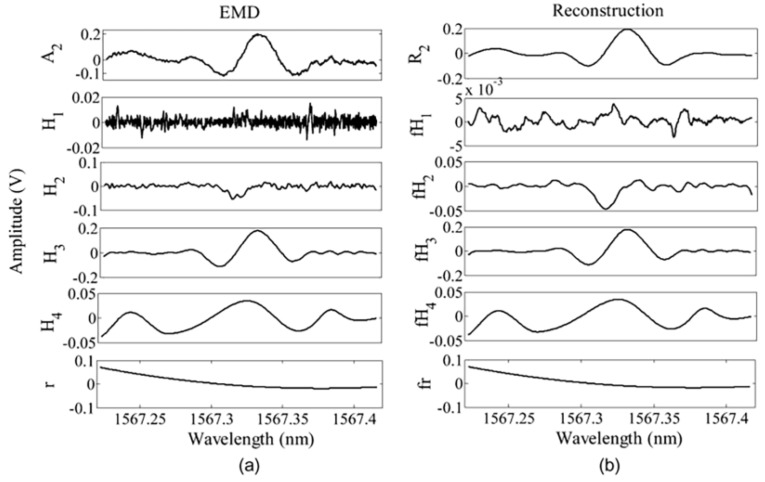
(**a**) Signal decomposition into IMF. (**b**) Reconstructed IMF after S–G filtering. This figure was obtained from reference [42].

The essence of EMD is using the thought of stationary time series (STS) to decompose a frequency irregular wave into multiple regular waves and residual waves (original waveform = Σ IMFs + residual wave). Each IMF must meet two conditions: (1) in the whole data set, the number of extrema and the number of zero crossings must either equal or differ at most by one; (2) at any point, the mean value of the envelope defined by the local maxima and the envelope defined by the local minima are zero. In the EMD-FCR algorithm, each IMF requires being filtered by S–G filter (Figure 7b) and then cross-correlation calculations to obtain the cross-correlation coefficients between the original signal and each filtered IMFs. 

Finally, each filtered IMF is weighed by its corresponding correlation coefficient and then added up to reconstruct a new signal. A portion of noise in original signal is removed by S–G filter. The remaining noise shows a low correlation with second harmonic signal so that it accounts for a small proportion of the reconstructed signal. Thus, the majority of the noise is suppressed.

**Figure 8:**
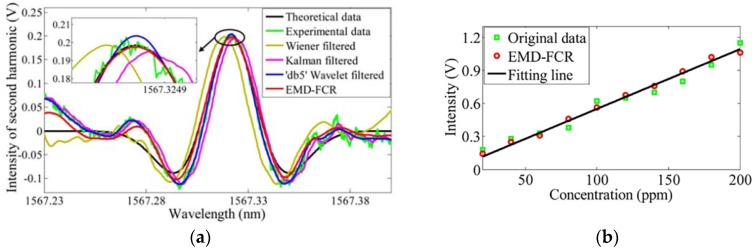
(**a**) Performance of different methods. (**b**) Relationship between the second harmonic intensity and gas concentration. This figure was obtained from [42].

The algorithm is assessed by simulation and experiment. In the two tests, EMD-FCR was compared with the Wiener filter, Kalman filter, and Wavelet filter. The results indicated that EMD-FCR performed best in both tests (Table 2 and Figure 8a). By this method, the SNR significantly improved from 7.32 dB to 14.31 dB, and the MDL decreased from 18 ppm to 2 ppm with SNR = 3 dB.

In further research, demodulation error experiments verified its reliability for extended (hour lomg) work. The errors of the second harmonic intensity after 50 min was only 2.113 × $10^{-5}$ V. Varying-concentration experiments indicated that the linear correlation coefficient of second harmonic intensity and gas concentration was improved from 0.93290 to 0.99297 by using the EMD-FCR algorithm.

#### 3.1.4. Summary of Denoise Algorithm

For signal denoising, WT is a powerful tool, which can achieve a high SNR. However, the WT algorithm relies on many parameters, such as setting the wavelet base and degree of decomposition, which are prone to introducing subjective errors. Adaptive S–G filter is an improved version of the SG filter whose window size and polynomial order vary with the local features of signals with high precision. Given that most commercial software libraries include a function for the S–G filter, this algorithm is easy to implement. However, due to its nature as a smoothing filter and its specialization in Gaussian noise, some S–G filter experiments shows that the method may not work well if the signal contains large fluctuations. The EMD-FCR algorithm is an improved algorithm based on empirical mode decomposition. The filtering principle of EMD-FCR is signal decomposition and reconstruction, which can deal with non-stationary signals well. The algorithm is also self-adaptive because this decomposition depends on the characteristic of the signal itself. However, this algorithm is not ideal. When dealing with scale-mutative signal, the EMD algorithm may suffer mode-mixing problems [68,69,70].

### 3.2. Interference Fringe

In some practical case [71,72], white noise removal algorithms cannot effectively solve the interference fringe problem, especially when the signal is severely affected. Ensuring the accuracy of signal extraction is hard. Therefore, after the noise reduction, the following algorithms can be used to remove the interference fringes. The Levenberg-Marquardt (L-M) nonlinear fitting and the semi-parametric interference-immune algorithm can perform this task, respectively, from the perspective of the time and frequency domains.

#### 3.2.1. L-M Nonlinear Fitting

L-M algorithm [73] is the most widely used nonlinear least-square algorithm, which uses gradient and iteration to find the largest or smallest value and then obtains the optimal solution of the requested parameters. Yan et al. [47] and Wagner et al. [74] used this algorithm for TDLAS curve-fitting. This algorithm converges fast and shows both the advantages of gradient method and Newton method [75,76,77]. However, one obvious drawback of L-M is that this iterative fitting requires a large amount of computation. Given the second-harmonic signal 2f, which is not a particularly complex function, has not too much parameter to be estimated, this method is usable for TDLAS signal processing. However, Yan et al. [47] mentioned that the L-M algorithm requires approximately 60,000 operations in a single iteration. Thus, adequate hardware support is indispensable to guarantee this algorithm will run well.

**Figure 9:**
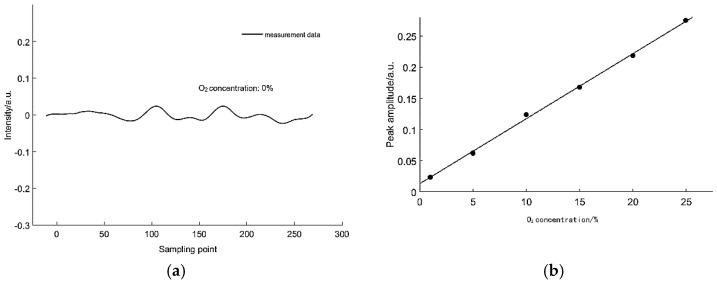
(**a**) Zero concentration fringe. (**b**) Relationship between the peak amplitude and gas concentration. This figure was adapted from [47].

The specific mathematical principles of the L-M algorithm are not elaborated here because of its extensive use. However, we simply introduce the general iteration steps:

(a) Select initial value $x_{0}$ and termination condition ε, and calculate $e_{0}=||Y-f\left(x_{0}\right)||$ and let step length $\lambda_{0}=10^{-3}$;
(b) Compute the Jacobian matrix $J_{x}^{k}$, and construct the incremental equations;
(c) Solve the incremental equation, and obtain $\Delta_{k+1}$;
(d) If $\left||Y-f\left(x_{0}\right)|\right|$ less than or equal to $e_{k}$, forward to e; else let $\lambda_{k-1}=10\lambda_{k}$ and go back to a;
(e) If $\left||\Delta_{k}|\right|$ less than ε, stop iteration and output the result; else let $\lambda_{k-1}=10\lambda_{k}$ and go back to b;

The baseline noise is measured first. As shown in Figure 9a, at a concentration of zero, the measured signal still shows pronounced fluctuation after smoothing filtration, which filtered out most of the high-frequency electronic noise.

In non-zero concentration experiments, the fitted and actual peak amplitudes show a 15% concentration error, which was the result of reducing the computational complexity of the L-M algorithm. After error correction, the measured nonlinearity between the gas concentration and the calculated concentration was 1.08%. This figure is 0.103% in the EMD algorithm experiment. However, because of the different measurement equipment and the different functional areas of the two algorithms, comparing these two values does not make much sense, that is, the two measurements may not use the same smoothing algorithm.

#### 3.2.2. Semi-Parametric Interference-Immune Algorithm

Michelucci and Venturini proposed a novel semi-parametric algorithm to eliminate the signal distortion and background fluctuation caused by interference [36]. This algorithm shows a significant effect for dealing with strong interference signals. Compared with some of the above time-domain algorithms, this method starts from the frequency domain to solve the problem that the time domain algorithm is not good at. For severely polluted signals, even though the signal amplitude is ten times smaller than the fringes, the time-domain waveform has been severely disturbed, and the conventional time-domain fitting makes it difficult to restore the signal itself. However, these disturbances are easily distinguished in frequency, independently of the amplitude of the interferences. In general, the desired signal in TDLAS system, like absorption peak and second-harmonic waveform, can be modeled using known linear type. Calculating the DFT of model function is easy. Therefore, the DFT of the measurement signal can be fitted to obtain the line type parameters, and entering the parameter is not needed in this algorithm. However, for general spectrum fitting algorithms, when the signal interference is too weak, the contribution of the undesired factors on the frequency spectrum is not obvious and this method is hard to be applied.

**Figure 10:**
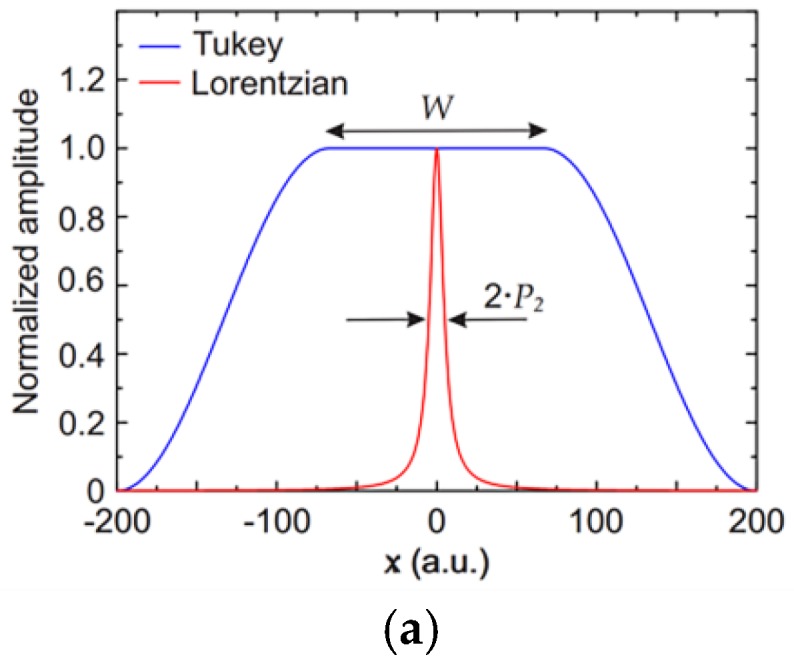

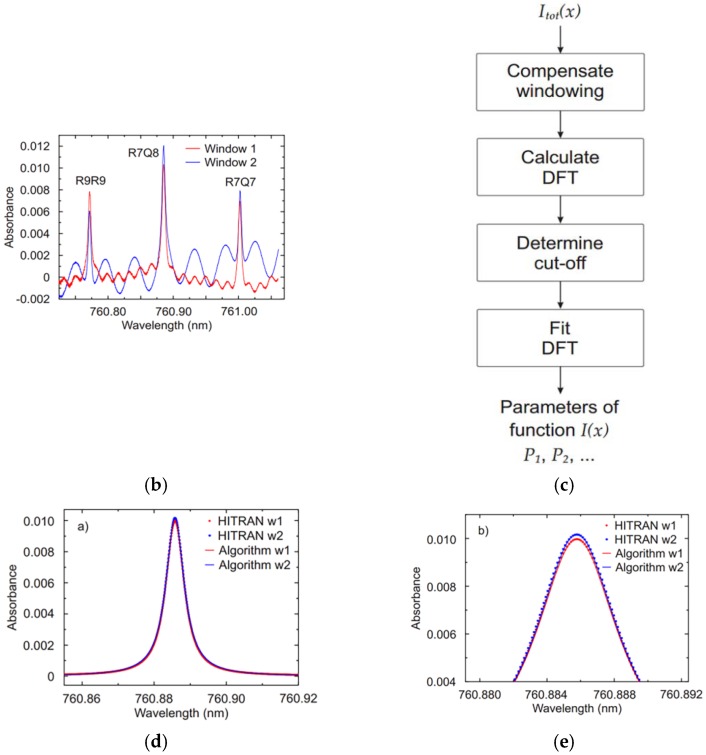
(**a**) Tukey window; (**b**) deliberately made interference fringe, (**c**) algorithm flowchart, (**d**) experimental result, (**e**) detail of the processed signal. This figure was adapted from [36].

This algorithm involves calculating the parameters by fitting the DFTs of model function and measured signal using the parameters to reconstruct the corresponding line shape. The steps of the algorithm are summarized in the figure. First, in order to improve the accuracy and reduce the window effect when measuring the signal DFT, the author chooses Tukey window (Figure 10a) is the compensation window [78,79] so that the signal decreases rapidly to zero on the sides. The next step is to determinate the optimal cut-off point $i_{0}$ to maximize the coefficient of determination $R^{2}$ obtained by fitting the DFT for i > $i_{0}$ to the functional form of the Fourier transform of the line shape. At every measurement, the $i_{0}$ is recalculated to guarantee that the algorithm will not be influenced by fringe changes in time, solving long-time stability problems arising from changes over time of the background, like thermal drift. Finally, DFT is fitted by using the DFT of model function to fit the DFT of the measured signal to obtain the parameters to determine the target signal. The algorithm flowchart is shown in Figure 10c.

In the simulation, the author simulates three background interferences to test the algorithm: periodic disturbance, weak disturbances of large FSR, and a complex disturbance with summing of 100 cosine functions. The result of simulation shows the discrepancy between the results obtained with the algorithm, and the expected values for the line parameters is less than 0.3%. As long as the background interference shows no fitting obstacle in the spectrum, the algorithm can perform signal extraction well, and this process is slightly affected by the interference amplitude.

Deliberately made interference fringes are utilized to test the practicality of the algorithm, and the measured signal is shown in Figure 10b. Two different windows interfere with two different intensity fringes. Despite the strong fluctuation, the extracted line shows a remarkable agreement with the expected curves from the HITRAN database [80], with deviation of the area of 0.1%. The experimental result is shown in Figure 10d,e.

These experiments show that the algorithm can effectively improve the system accuracy in the case of strong interference and solve the background fluctuation of the signal in a targeted manner. On the other hand, additional experiments remain to be performed to test the performance of this algorithm under the interference of other features.

#### 3.2.3. Summary of Interference Fringe Processing Algorithm

For interference fringe problem, L-M nonlinear fitting and semi-parametric interference-immune algorithm are two solutions discussed in this paper. The L-M algorithm fits the signal in the time domain and is a widely utilized nonlinear least-squares method. This algorithm offers the advantages of both the Newton method and gradient method and fast convergence. Nevertheless, this iterative fitting requires the device to possess a high computational power. The semi-parametric interference-immune algorithm is a spectral fitting algorithm that can cope with the difficult situation of many time-domain analysis and presents strong immunity to strong optical interference signals. The signal extraction is independent from the amplitude of the interference. This method requires that the interference fringes of the measurement signal can be easily resolved in the spectrum, if the interference fringes are small, time-domain fitting can be performed directly and does not require spectrum analysis.

### 3.3. Baseline Drift

Background correction is required before signal fitting, otherwise the background will produce a large error for some fitting algorithms. In the following sections, two background correction strategies are introduced. These strategies adopt an iterative method to maximize the real baseline position. These strategies may be based on some algorithmic improvements with high reference value.

#### 3.3.1. Advanced Integrative (AI) Algorithm

The AI algorithm proposed by Skrotzki et al. [51] is a modified fitting algorithm for the drawback of the integrative evaluation method, which calculates the molecular concentration by the integral area of the absorption line. Thus, the baseline error is made close to zero by fitting the no-absorption area and multiple iterations to improve the accuracy of the calculation results. An important feature is that the AI fitting algorithm is restricted to the evaluation of single absorption lines with precomputed line width. This feature suffers from limitations but exhibits a very fast reaction rate, and the fitting process does not dependent on appropriately chosen start values for the initialization, indicating its advantages in terms of robustness.

In particular, the authors compared it with the L-M algorithm and proved that the algorithm achieves similar accuracy as the L-M algorithm under proper application conditions, and the speed is 3–4 times faster than the L-M algorithm compared with the huge computational load of the latter. The AI method can be applied to embedded systems with limited computing power. In conclusion, this algorithm is an alternative for dealing with single absorption peak fitting in TDLAS systems.

Before introducing the principle, emphasizing the three assumptions and prerequisites for applying this algorithm is necessary: (1) the incident intensity $I_{0}$ (a parameter in Beer-Lambert law) is sufficiently known; (2) measurement signal is directly given in wavenumber domain; (3) only a single line absorption spectrum is considered.

**Figure 11:**
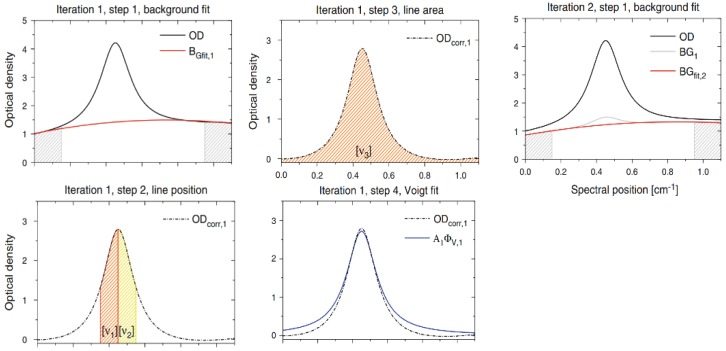
Steps of advanced integrative algorithm. This figure was obtained from [51].

Each iteration involves four steps (Figure 11). In Step 1, a polynomial fit is applied to the flanks of the absorption line signal to correct the background. In Step 2, the absorption line position $m_{0}$ is determined to retrieve the full absorption line profile. In Step 3, the line area obtained by integrate is corrected for the area within the flanks of the absorption line that are not covered by $\left[v_{3}\right]$ (shown in the figure). In Step 4, Voigt fit is used to obtain a good line shape that is approximate to the actual background.

In the second and subsequent iterations, Step 1 aims to fit the previous iteration by the same method, and the other steps are performed in the same manner discussed in the procedure described above. After multiple iterations, the precision of line area and line position continues to increase.

In this experiment, six iterations were necessary to fulfill the terminating conditions the author has chosen, yielding a fit precision of the line area $A_{6}$ of at least $10^{-3}$ and of the line position $v_{6}$ of at least $10^{-4}$, respectively. Each iteration’s results are shown in Table 3.

**Figure 12:**
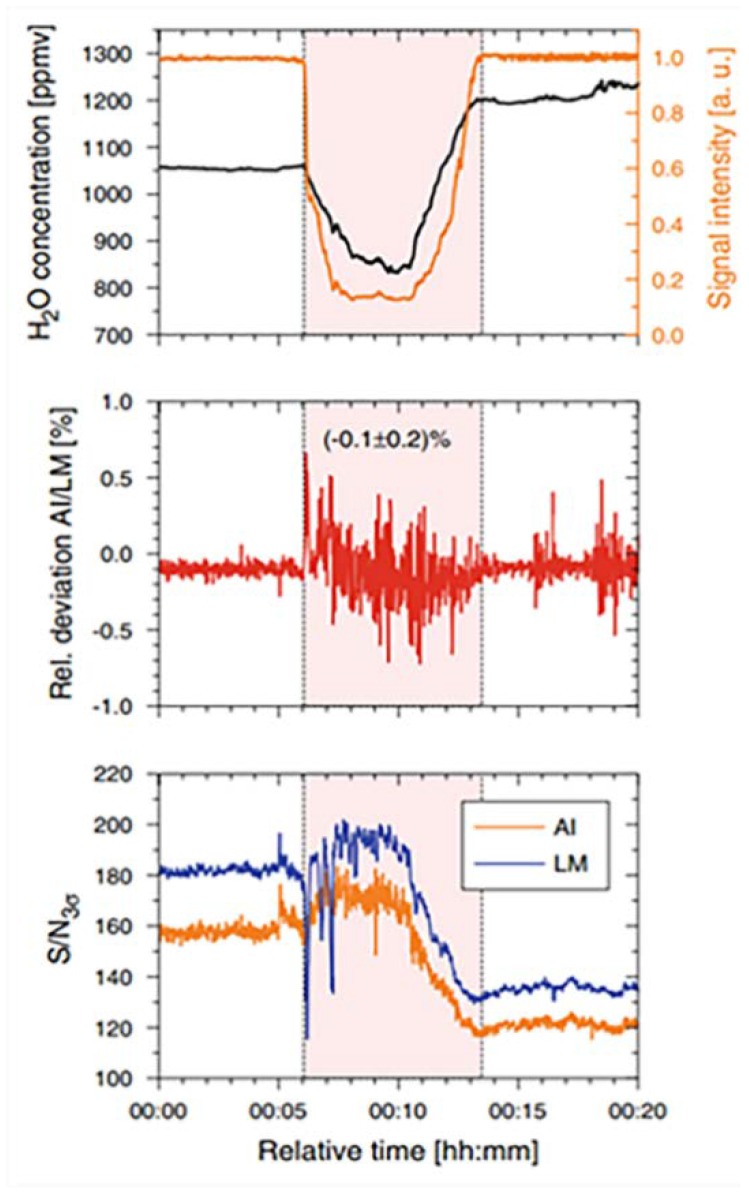
Comparison of AI fit and LM fit with relative time. This figure was obtained from [51].

The AI and L-M algorithms were used to compare the water vapor measurement experiments. The average relative deviation of the two algorithms was 0.1 ± 0.2%, and the peak relative deviation was maintained within the range of ±0.7%. Figure 12 shows the dynamic response of the relative deviation and SNR with the dynamic variation of $H_{2}O$ concentration. Moreover, typical computational times obtained for the AI algorithm were 100–200 μs for the full evaluation of an absorption line profile. On the other hand, if the absorption line profile in the measurement signal was very unsatisfactory, it may not ideally converge. In the article, the authors summarize the characteristics of the two algorithms in terms of stability, speed, and flexibility, as shown in the figure, which can be a reference in practical applications.

#### 3.3.2. Wavelet-Based Method for Baseline Drift

An AI algorithm calculates the background line by fitting the no absorption flanks. However, for poor-quality spectra, distinguishing the no absorption area using direct visual inspection (DVI) is hard. A new strategy was proposed by Li et al. [52], using wavelet decomposition and iteration to remove drift background. The application of WT for TDLAS signal denoising has been introduced, but the above studies tend to solve high-frequency noise, such as white Gaussian noise. This method uses WT based on the optimal wavelet pairs to find baseline and uses iteration to determine the precise location. In addition to the solution to the baseline drift, the strategy of separating the process of denoising and removing background is also meaningful. Unlike the block threshold strategy [81], this method uses different wavelet and decomposition levels to deal with noise and baseline. The characteristics of the two types of interference are considered and which of the two types shows a strong sense of reference is discussed.

**Figure 13:**
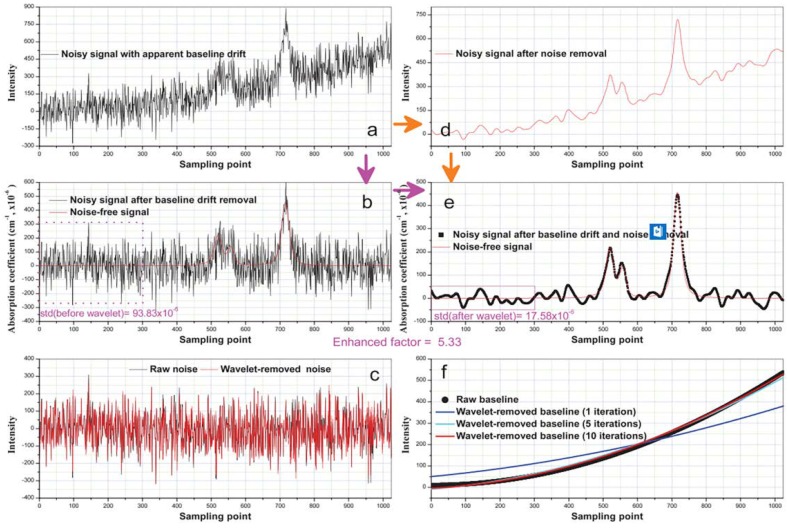
Nonlinear baseline correction and denoising using DWT. (**a**) Noisy signal with apparent baseline drift; (**b**) baseline drift removed signal from (**a**) and noise-free signal; (**c**) raw noise and wavelet-removed noise; (**d**) denoised signal from (**a**); (**e**) baseline drift removed signal from (**d**) and noise-free signal; (**f**) raw baseline and wavelet-removed baseline. This figure was obtained from [52].

In this method, denoising is separate from baseline removal regardless of their order. To remove the background, it is found that the wavelets bior2.2 or bior3.3 are good candidates for denoising TDLAS signals. A higher decomposition level than the optimal decomposition level for denoising was performed first. All detail coefficients were set to zero, and the approximation coefficients were used to reconstruct the signal. In this manner, a main background is obtained. The raw signal from the main background is subtracted, decomposed, and reconstructed. The iteration is repeated until the background reaches a precision calculated by root mean square error. Typically, this procedure is finished within 10 iterations. After removing the background, wavelet Daubechies7 is used for denoising. Conversely, an optimal decomposition level than the high decomposition level for denoising was first performed, and the best decomposition level between 5–7 was selected. The noise was then removed by decomposition and reconstruction. Figure 13 shows that the simulation results of the algorithm were very successful, the background was effectively corrected while preserving primary useful information, and the SNR was significantly improved.

**Figure 14:**
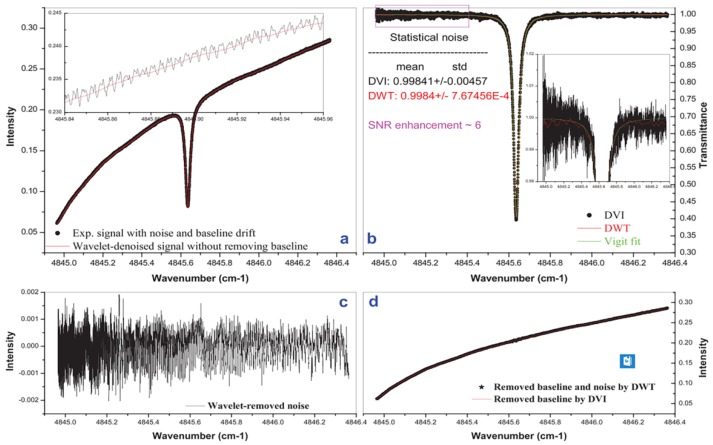
(**a**) Experimental spectrum of CO_2_ (pressure = 140 mbar; path length = 4800 cm; mixing ratio = 1.0%; temperature = 292.95 K) and wavelet–denoised signal; (**b**) Baseline drift removed transmittance signals using DVI and DWT, as well as the Voigt fit; (**c**) DWT removed noise; (**d**) DWT removed baseline with noise and DVI removed baseline. Parts of the “baselines” are expanded for clarity and statistical noises are also provided in the inset. This figure was obtained from [52].

The CO_2_ absorption experiment, which is shown in Figure 14, demonstrates the effectiveness of this algorithm for solving baseline drift problems. The calculated SNR in DVI and DWT are 131.3 and 781.8, respectively.

#### 3.3.3. Summary of Background Removal Algorithms

In background correction, both the AI algorithm and the wavelet-based schemes improve the accuracy by introducing an iteration process. The AI algorithm corrects the background iteration through the absorption line area. The algorithm is very lightweight and suitable for solving the simple case of a single absorption peak, which poses the advantages of small calculation and fast speed. The latter uses decomposed wavelet background correction and iteration. In this strategy, the authors separate the background correction from denoising and use different wavelet and decomposition levels to process the background and the noise, respectively, which demonstrates flexible utilization of WT.

## 4. Conclusions

When dealing with TDLAS signals, optical factors, electronic factors, and the nature of the semiconductor lasers can cause disturbances. These common problems are summarized in three models: signal denoising, interference fringes, and background correction. In the above article, we have reviewed and compared some effective algorithms based on resent research works. Representative experiments were presented to evaluate the performance both qualitatively and quantitatively.

In essence, these signal problems are interferences superimposed on the original signal. These interferences are classified into three noise models for reduction due to the differences in spectral characteristic. Electrical white noise is a multiple frequency signal with a small amplitude, whereas interference fringes show concentrated frequency and large amplitude, and baseline drift is a ramp signal close to DC. Therefore, different strategies must be selected to deal with different signal models. For example, the adaptive S–G algorithm, which utilizes shift windows, can eliminate high-frequency/low-frequency well. By contrast, low-frequency and high-amplitude interference noise is difficult to remove with smoothing algorithms, but interference fringes are easy to process in the frequency domain by using a semi-parametric interference-immune algorithm. Nevertheless, the strategy involved in the algorithm must not be limited to the algorithm itself, like the schemes of correlation coefficient weight method, iteration, adaptive improvement, and problem decomposition, which can offer a foothold to solve any problem.

In comparison, signal decomposition and reconstruction-based algorithms, such as WT and EMD-FCR, can partly deal with all the three noise models because of their properties, such as multiscalability. When using WT, the selected parameters show remarkable effects. Therefore, when comparing algorithms, the details of experiments should be given particular importance. In future research, we expect that additional flexible strategies of signal decomposition and reconstruction algorithms will be developed to broaden their application for a wider variety of noise models.

## Figures and Tables

**Table 1:** Comparison between the sensing performances under the cases of with and without WD use. Accu: accuracy; MDL: minimum detection limit; RT: response time; AD: Allan deviation. This table was obtained from reference [40].

	MDL (ppm)	Non-Averaged Detection Range on 4 ppm Gas Sample (ppm)	Accu (%)	AD (ppm)
Using WD	1	3–5	<3.8% (C > 4 ppm)	0.08 (τ = 500 s) 0.35 (τ = 30 s)
Without using WD	4	2.6–5.5	<6.2% (C > 4 ppm)	0.13 (τ = 500 s) 0.46 (τ = 30 s)

**Table 2:** SNR and residual sum of squares (SSR) of different filters. This table was obtained from reference [42].

Filter	Wiener	Kalman	Wavelet	EMD-FCR
${\mathrm{SNR}}_{1}\;(\mathrm{dB})$	14.17	11.21	13.26	14.82
${\mathrm{SSR}}_{1}\;(V^{2})$	0.0287	0.3010	0.0027	0.0014
${\mathrm{SNR}}_{2}\;(\mathrm{dB})$	14.02	11.81	13.96	14.31
${\mathrm{SSR}}_{2}\;(V^{2})$	0.9859	0.7523	0.5943	0.2538

**Table 3:** Evolution of the relative deviation of absorption line area A*_i_* and position mi from the prescribed ‘true’ values together with the signal-to-noise ratio $S/N_{3\sigma ,i}$ for each iteration of the AI fit. This table is from [41].

Iteration *i*	Rel. Dev. Line Area	Rel. Dev. Line Position	$S/N_{3\sigma ,i}$
1	0.118	4 × 10^−4^	12
2	0.038	3 × 10^−4^	38
3	0.012	3 × 10^−4^	119
4	0.004	2 × 10^−4^	339
5	0.001	2 × 10^−4^	726
6	<0.001	1 × 10^−4^	1060

## References

[1] Cassidy D.T., Reid J. (1982). Atmospheric pressure monitoring of trace gases using tunable diode lasers. Appl. Opt..

[2] Wang W., Lv Y. (2018). The principal, preparation and application of quantum cascade laser. Laser J..

[3] Nikodem M., Wysocki G. (2012). Chirped laser dispersion spectroscopy for remote open-path trace-gas sensing. Sensors.

[4] Li M., Bai F. (2018). Design of High Sensitivity Infrared Methane Detector Based on TDLAS-WMS. Laser J..

[5] Kluczynski P., Jahjah M., Nähle L., Axner O., Belahsene S., Fischer M., Koeth J., Rouillard Y., Westberg J., Vicet A. (2011). Detection of acetylene impurities in ethylene and polyethylene manufacturing processes using tunable diode laser spectroscopy in the 3-μm range. Appl. Phys. B.

[6] Zhang L., Cui X. (2014). The Design of Carbon Monoxide Detector Based on Tunable Diode Lasers Absorption Spectroscope. Laser J..

[7] Werle P. (1998). A review of recent advances in semiconductor laser based gas monitors. Spectrochim. Acta Part A Mol. Biomol. Spectrosc..

[8] Werle P., Slemr F., Maurer K., Kormann R., Mücke R., Jänker B. (2002). Near-and mid-infrared laser-optical sensors for gas analysis. Opt. Lasers Eng..

[9] Wang F., Cen K., Li N., Jeffries J.B., Huang Q., Yan J., Chi Y. (2010). Two-dimensional tomography for gas concentration and temperature distributions based on tunable diode laser absorption spectroscopy. Meas. Sci. Technol..

[10] Kurtz J., Aizengendler M., Krishna Y., Walsh P., O’Byrne S.B. Flight test of a rugged scramjet-inlet temperature and velocity sensor. Proceedings of the 53rd AIAA Aerospace Sciences Meeting.

[11] Wang F., Wu Q., Huang Q., Zhang H., Yan J., Cen K. (2015). Simultaneous measurement of 2-dimensional H_2_O concentration and temperature distribution in premixed methane/air flame using TDLAS-based tomography technology. Opt. Commun..

[12] Buchholz B., Afchine A., Ebert V. (2014). Rapid, optical measurement of the atmospheric pressure on a fast research aircraft using open-path TDLAS. Atmos. Meas. Tech..

[13] Lins B., Zinn P., Engelbrecht R., Schmauss B. (2010). Simulation-based comparison of noise effects in wavelength modulation spectroscopy and direct absorption TDLAS. Appl. Phys. B.

[14] Chen K., Mei M. (2014). Detection of Gas Concentrations Based on Wireless Sensor and Laser Technology. Laser J..

[15] Mueller H.G., Weber J., Hornsby B.W.Y. (2006). The effects of digital noise reduction on the acceptance of background noise. Trends Amplif..

[16] Misiti M., Misiti Y., Oppenheim G., Poggi J.-M. (2010). Wavelets and their applications. Int. J. Imaging Syst. Technol..

[17] Li J., Yu B., Zhao W., Chen W. (2014). A review of signal enhancement and noise reduction techniques for tunable diode laser absorption spectroscopy. Appl. Spectrosc. Rev..

[18] Zhang K., Zhang L., Zhao Q., Liu S., Chen S., Wu Y., Wang K., Yang X. (2017). Application of digital quadrature lock-in amplifier in TDLAS humidity detection. Opt. Spectrosc. Imaging Int. Soc. Opt. Photonics.

[19] Mohammad I.L., Anderson G.T., Chen Y. (2014). Noise estimation technique to reduce the effects of 1/f noise in Open Path Tunable Diode Laser Absorption Spectrometry. Int. Soc. Opt. Photonics.

[20] Chighine A., Fisher E., Wilson D., Lengden M., Johnstone W., McCann H. An FPGA-based lock-in detection system to enable Chemical Species Tomography using TDLAS. Proceedings of the 2015 IEEE International Conference on Imaging Systems and Techniques (IST).

[21] Tu G., Dong F., Wang Y., Culshaw B., Zhang Z., Pang T., Xia H., Wu B. (2015). Analysis of random noise and long-term drift for tunable diode laser absorption spectroscopy system at atmospheric pressure. IEEE Sens. J..

[22] He Q., Dang P., Liu Z., Zheng C., Wang Y. (2017). TDLAS–WMS based near-infrared methane sensor system using hollow-core photonic crystal fiber as gas-chamber. Opt. Quantum Electron..

[23] Frish M., Wainner R., Laderer M., Parameswaran K., Sonnenfroh D., Druy M. (2011). Precision and accuracy of miniature tunable diode laser absorption spectrometers. Proc. SPIE.

[24] Knight J., Birks T., Cregan R., Russell P.S.J., De Sandro P. (1998). Large mode area photonic crystal fibre. Electron. Lett..

[25] Dong L., Tittel F.K., Li C., Sanchez N.P., Wu H., Zheng C., Yu Y., Sampaolo A., Griffin R.J. (2016). Compact TDLAS based sensor design using interband cascade lasers for mid-IR trace gas sensing. Opt. Express.

[26] Wang F., Chang J., Wang Q., Wei W., Qin Z. (2017). TDLAS gas sensing system utilizing fiber reflector based round-trip structure: Double absorption path-length, residual amplitude modulation removal. Sens. Actuators A Phys..

[27] Shao L., Yan R., Li X., Liu Y. (2014). From heuristic optimization to dictionary learning: A review and comprehensive comparison of image denoising algorithms. IEEE Trans. Cybern..

[28] Gupta K., Gupta S. (2013). Image denoising techniques-a review paper. IJITEE.

[29] Werle P., Slemr F. (1991). Signal-to-noise ratio analysis in laser absorption spectrometers using optical multipass cells. Appl. Opt..

[30] Masiyano D., Hodgkinson J., Tatam R.P. (2008). Use of diffuse reflections in tunable diode laser absorption spectroscopy: Implications of laser speckle for gas absorption measurements. Appl. Phys. B.

[31] Bomse D.S., Stanton A.C., Silver J.A. (1992). Frequency modulation and wavelength modulation spectroscopies: Comparison of experimental methods using a lead-salt diode laser. Appl. Opt..

[32] Hennig O., Strzoda R., Mágori E., Chemisky E., Tump C., Fleischer M., Meixner H., Eisele I. (2003). Hand-held unit for simultaneous detection of methane and ethane based on NIR-absorption spectroscopy. Sens. Actuators B Chem..

[33] Le Barbu T., Vinogradov I., Durry G., Korablev O., Chassefière E., Bertaux J.-L. (2006). TDLAS a laser diode sensor for the in situ monitoring of H_2_O, CO_2_ and their isotopes in the Martian atmosphere. Adv. Space Res..

[34] Weibring P., Richter D., Fried A., Walega J., Dyroff C. (2006). Ultra-high-precision mid-IR spectrometer II: System description and spectroscopic performance. Appl. Phys. B.

[35] Buchholz B., Kühnreich B., Smit H., Ebert V. (2013). Validation of an extractive, airborne, compact TDL spectrometer for atmospheric humidity sensing by blind intercomparison. Appl. Phys. B.

[36] Michelucci U., Venturini F. (2017). Novel semi-parametric algorithm for interference-immune tunable absorption spectroscopy gas Sensing. Sensors.

[37] Wang J., Yu D., Ye H., Yang J., Ke L., Han S., Gu H., Chen Y. (2011). Applications of optical measurement technology in pollution gas monitoring at thermal power plants. Proc. SPIE.

[38] Reid J., Labrie D. (1981). Second-harmonic detection with tunable diode lasers—Comparison of experiment and theory. Appl. Phys. B.

[39] Xia H., Dong F.-Z., Zhang Z.-R., Tu G.-J., Pang T., Wu B., Wang Y. (2010). Signal analytical processing based on wavelet transform for tunable diode laser absorption spectroscopy. Proc. SPIE.

[40] Zheng C.-T., Ye W.-L., Huang J.-Q., Cao T.-S., Lv M., Dang J.-M., Wang Y.-D. (2014). Performance improvement of a near-infrared CH_4_ detection device using wavelet-denoising-assisted wavelength modulation technique. Sens. Actuators B Chem..

[41] Li J., Deng H., Li P., Yu B. (2015). Real-time infrared gas detection based on an adaptive Savitzky–Golay algorithm. Appl. Phys. B.

[42] Meng Y., Liu T., Liu K., Jiang J., Wang R., Wang T., Hu H. (2014). A modified empirical mode decomposition algorithm in TDLAS for gas detection. IEEE Photonics J..

[43] Hodgkinson J., Tatam R.P. (2012). Optical gas sensing: A review. Meas. Sci. Technol..

[44] Hartmann A., Strzoda R., Schrobenhauser R., Weigel R. (2014). Ultra-compact TDLAS humidity measurement cell with advanced signal processing. Appl. Phys. B.

[45] Cao J.N., Wang Z., Zhang K.-K., Yang R., Wang Y. Etalon effects analysis in tunable diode laser absorption spectroscopy gas concentration detection system based on wavelength modulation spectroscopy. Proceedings of the Symposium on Photonics and Optoelectronics.

[46] Masiyano D., Hodgkinson J., Schilt S., Tatam R.P. (2009). Self-mixing interference effects in tunable diode laser absorption spectroscopy. Appl. Phys. B.

[47] Yan J., Zhai C., Wang X., Huang W. (2015). The research of oxygen measurement by TDLAS based on Levenberg-Marquardt nonlinear fitting. Spectrosc. Spectr. Anal..

[48] Webster C.R. (1985). Brewster-plate spoiler: A novel method for reducing the amplitude of interference fringes that limit tunable-laser absorption sensitivities. JOSA B.

[49] Huang N.E., Shen Z., Long S.R., Wu M.C., Shih H.H., Zheng Q., Yen N.-C., Tung C.C., Liu H.H. (1998). The empirical mode decomposition and the Hilbert spectrum for nonlinear and non-stationary time series analysis. Proc. Math. Phys. Eng. Sci..

[50] Xie Q., Li J., Gao X., Jia J. (2009). Real time infrared gas detection based on a modified EMD algorithm. Sens. Actuators B Chem..

[51] Skrotzki J., Habig J.C., Ebert V. (2014). Integrative fitting of absorption line profiles with high accuracy, robustness, and speed. Appl. Phys. B.

[52] Li J., Yu B., Fischer H. (2015). Wavelet transform based on the optimal wavelet pairs for tunable diode laser absorption spectroscopy signal processing. Appl. Spectrosc..

[53] Werle P., Mücke R., Slemr F. (1993). The limits of signal averaging in atmospheric trace-gas monitoring by tunable diode-laser absorption spectroscopy (TDLAS). Appl. Phys. B.

[54] Werle P.W., Scheumann B., Schandl J. (1994). Real-time signal-processing concepts for trace-gas analysis by diode-laser spectroscopy. Opt. Eng..

[55] Leleux D., Claps R., Chen W., Tittel F., Harman T. (2002). Applications of Kalman filtering to real-time trace gas concentration measurements. Appl. Phys. B.

[56] Coifman R.R., Meyer Y., Wickerhauser V. (1992). Wavelet analysis and signal processing. Wavelets and Their Applications.

[57] Tsatsanis M.K., Giannakis G.B. Multirate filter banks for code-division multiple access systems. Proceedings of the International Conference on Acoustics, Speech, and Signal Processing, ICASSP-95.

[58] Li J., Parchatka U., Fischer H. (2012). Applications of wavelet transform to quantum cascade laser spectrometer for atmospheric trace gas measurements. Appl. Phys. B.

[59] Duan H., Gautam A., Shaw B.D., Cheng H.H. (2009). Harmonic wavelet analysis of modulated tunable diode laser absorption spectroscopy signals. Appl. Opt..

[60] Savitzky A., Golay M.J. (1964). Smoothing and differentiation of data by simplified least squares procedures. Anal. Chem..

[61] Madden H.H. (1978). Comments on the Savitzky-Golay convolution method for least-squares-fit smoothing and differentiation of digital data. Anal. Chem..

[62] Czarnecki M.A. (2015). Resolution enhancement in second-derivative spectra. Appl. Spectrosc..

[63] Boudraa A.-O., Cexus J.-C. (2007). EMD-based signal filtering. IEEE Trans. Instrum. Meas..

[64] Pines D., Salvino L. (2006). Structural health monitoring using empirical mode decomposition and the Hilbert phase. J. Sound Vib..

[65] Poungponsri S., Yu X.-H. (2013). An adaptive filtering approach for electrocardiogram (ECG) signal noise reduction using neural networks. Neurocomputing.

[66] Lingfang S., Yechi W. (2012). Soft-sensing of oxygen content of flue gas based on mixed model. Energy Procedia.

[67] Kopsinis Y., McLaughlin S. (2009). Development of EMD-based denoising methods inspired by wavelet thresholding. IEEE Trans. Signal Process..

[68] Tang B., Dong S., Song T. (2012). Method for eliminating mode mixing of empirical mode decomposition based on the revised blind source separation. Signal Process..

[69] Hu X., Peng S., Hwang W.-L. (2012). EMD revisited: A new understanding of the envelope and resolving the mode-mixing problem in AM-FM signals. IEEE Trans. Signal Process..

[70] Torres M.E., Colominas M.A., Schlotthauer G., Flandrin P. A complete ensemble empirical mode decomposition with adaptive noise. Proceedings of the IEEE International Conference on Acoustics, Speech and Signal Processing (ICASSP).

[71] Werle P. (1995). Laser excess noise and interferometric effects in frequency-modulated diode-laser spectrometers. Appl. Phys. B.

[72] Hansen P., Pereyra V., Scherer G. (2004). Nonlinear Least Squares Problems. http://www.imm.dtu.dk/pcha/LSDF/NonlinDataFit.pdf.

[73] Ranganathan A. (2004). The levenberg-marquardt algorithm. Tutoral LM Algorithm.

[74] Wagner S., Klein M., Kathrotia T., Riedel U., Kissel T., Dreizler A., Ebert V. (2012). Absolute, spatially resolved, in situ CO profiles in atmospheric laminar counter-flow diffusion flames using 2.3 μm TDLAS. Appl. Phys. B.

[75] Bertsekas D.P. (1999). Nonlinear Programming.

[76] Wedderburn R.W. (1974). Quasi-likelihood functions, generalized linear models, and the Gauss—Newton method. Biometrika.

[77] Hartley H.O. (1961). The modified Gauss-Newton method for the fitting of non-linear regression functions by least squares. Technometrics.

[78] Tukey J.W. (1967). An introduction to the calculations of numerical spectrum analysis. Spectra Anal. Time.

[79] Harris F.J. (1978). On the use of windows for harmonic analysis with the discrete Fourier transform. Proc. IEEE.

[80] Rothman L.S., Gordon I.E., Babikov Y., Barbe A., Benner D.C., Bernath P.F., Birk M., Bizzocchi L., Boudon V., Brown L.R. (2013). The HITRAN2012 molecular spectroscopic database. J. Quant. Spectrosc. Radiat. Transf..

[81] Ramos P.M., Ruisánchez I. (2005). Noise and background removal in Raman spectra of ancient pigments using wavelet transform. J. Raman Spectrosc..

